# Dissatisfaction about body image and associated factors: a study of young undergraduate students

**DOI:** 10.31744/einstein_journal/2019AO4642

**Published:** 2019-08-14

**Authors:** Laleska Pâmela Rodrigues da Silva, Ana Rita de Oliveira Tucan, Elisana Lima Rodrigues, Patrícia Vieira Del Ré, Priscila Milene Angelo Sanches, Deise Bresan

**Affiliations:** 1 Faculdade de Ciências Farmacêuticas, Alimentos e Nutrição, Universidade Federal de Mato Grosso do Sul, Campo Grande, MS, Brazil

**Keywords:** Self concept, Nutritional status, Body image, Body weight, Students

## Abstract

**Objective:**

To verify the prevalence of dissatisfaction with body image and its association with socio-demographic, economic, and anthropometric variables, as well as levels of physical activity among undergraduate students.

**Methods:**

A cross-sectional study was conducted with 348 undergraduate students at the dining hall of a public Brazilian university located in the state of Mato Grosso do Sul. Body image perception was evaluated using the Silhouette Matching Task. The other variables assessed were sex, age, marital status, housing conditions, socioeconomic class, weight, height, waist circumference and physical activity levels. Multinomial logistic regression was performed to verify the association between the dependent and independent variables.

**Results:**

Of the interviewees, 55.7% were men. The prevalence of dissatisfaction with body image was 59.8% among men and 55.2% among women. Dissatisfaction for being overweight, between men and women, was higher in overweight individuals when compared to normal weight individuals, according to the body mass index, and also higher in those at risk for cardiovascular disease when compared to those who were not at risk. The dissatisfaction for being thin was higher among women with low weight when compared to normal weight women, according to body mass index. There was no association between dissatisfaction for being thin and the variables analyzed among men.

**Conclusion:**

The prevalence of dissatisfaction with body image was observed in more than half of the individuals evaluated and is associated with nutritional status. Knowing the consequences of dissatisfaction with body image helps highlight the need for intervention strategies to avoid the practice of unhealthy behaviors.

## INTRODUCTION

As from the middle of the 20^th^ Century, Brazil began to see changes in the profile of morbidity and mortality of the population. Transformation in eating and physical activity habits have been responsible, in part, for the process of nutritional transition in the Brazilian population, with a decrease of malnutrition and increase in prevalence of overweight and obesity among adults.^(^^1^^,^^2^^)^ Currently, half of the adults in the country present with excess weight, and 18.9% are obese.^(^^3^^)^

At the same time in which one experiences a society of excesses, where many food products are available for purchase, with a wide availability of processed and ultraprocessed food, paradoxically one deals with thousands of messages to avoid them, in a society that promotes perpetual satisfaction and – simultaneously - stricter slimness.^(^^4^^)^ The pressure exerted by nutritional discourses and by marketing of the body in the construction of body image is excessive, and in the last decades, an ever increasing number of people have been manifesting dissatisfaction with the bodily shapes, abstaining from certain foods and often using inappropriate mechanisms of weight loss.^(^^4^^)^

Perception of one's body image is a complex phenomenon that involves cognitive, emotional, social, and cultural aspects. It is influenced by the dynamic interactions between the being and the environment in which they live, based on the feeling that a person has regarding body size and form.^(^^5^^)^ Beauty standards, strongly influenced by media and strengthened by society, have required ever more lean anthropometric profiles, leading to concern with body image even with a nutritional status within parameters considered adequate for the age range and sex.^(^^6^^,^^7^^)^

Therefore, it is fundamental to recognize the frequency of body dissatisfaction in the Brazilian population and what the possible associated factors are, since the same data produced can serve as a basis for future policies and programs in the field of healthcare, with the purpose of encouraging healthy eating habits, minimizing the pressure exerted by society relative to the body, and preventing the consequences triggered by discontent.

## OBJECTIVE

To verify the prevalence of dissatisfaction with body image and its association with sociodemographic, economic, and anthropometric variables, and physical activity of undergraduate students of both sexes.

## METHODS

This is a cross-sectional study, conducted with 348 undergraduate students who are clients of a dining hall of a public university of the State of Mato Grosso do Sul. To calculate the sample, Open Epi (version 3.0.1) software was used, with a target population of 1,800 individuals (daily average of diners), expected prevalence of 50.0%, margin of error of 5 percentage points, and an alpha error of 5.0%. The students were selected in the line of the restaurant by systematic sampling (out of every 15 individuals, the 15^th^ person was selected). The inclusion criteria were to frequent the restaurant at least three times a week, age equal to or more than 18 years and less than 30 years, and not be pregnant. The research was approved by the Human Research Ethics Committee (opinion 867.367, CAAE: 37575114.1.0000.0021), and all participants signed the Informed Consent Form (ICF). Data collection was done during five consecutive days in November 2015, by a previously trained team, and after execution of a pilot project. Data were collected on body image perception, nutritional status, physical activity, socioeconomic class, and sociodemographic variables (sex, age, living conditions, and marital status).

For evaluation of body perception, the Silhouette Matching Task (SMT) or Evaluation Test of Body Image was used,^(^^8^^)^ adapted and validated for Brazilian adults,^(^^9^^)^ which consists of a scale with nine figures of visual silhouettes. The individual was instructed to choose one of the figures corresponding to the self-evaluated body silhouette and another desired body silhouette. When there was a difference between the chosen silhouettes, the individual was considered dissatisfied with his/her own body image. Dissatisfaction occurred when the desired silhouette was smaller than the self-evaluated, and dissatisfaction with slimness when the desired silhouette was larger than the self-evaluated.

To determine nutritional status, the following variables were collected: body weight, height, and waist circumference (WC). These three parameters were measured based on the Lohman et al., protocol.^(^^10^^)^ To measure weight, portable digital platform-style scales were use, with 150kg capacity and 100g precision. Height was checked with a flexible and inextensible measuring tape, with 1mm precision. The tape was affixed to the wall at an initial height of 50cm. Waist circumference was measured with a flexible and inextensible metal measuring tape, with 1mm precision. After obtaining the body weight and height data, the body mass index (BMI) was calculated and classified as per the cut-off points proposed by the World Health Organization (WHO).^(^^11^^)^ Waist circumference was classified by criteria proposed by WHO.^(^^12^^)^

The level of physical activity was evaluated by the International Physical Activity Questionnaire (IPAQ), short version.^(^^13^^)^ Classification followed the WHO criteria, which considered as physically active that individual who performs at least 150 minutes a week of moderate physical activity or 75 minutes of vigorous physical activity, in sessions of at least 10 minutes duration, with no determination of weekly frequency.^(^^14^^)^

To determine the socioeconomic class, we applied the Brazilian Criterion of Economic Classification, of the Brazilian Association of Research Companies (ABEP - *Associação Brasileira de Empresas de Pesquisa*).^(^^15^^)^

Data were tabulated with EpiData 3.1 (EpiData Assoc., Odense, Denmark). Descriptive statistics were determined by relative frequency and 95% confidence interval (95%CI). Multinomial logistic regression was conducted to verify the association between the dependent variable (body image perception categorized into satisfied with body image, dissatisfied with excess weight, and dissatisfied with skinniness), and the independent variables (age, marital status, living conditions, socioeconomic class, BMI, WC, and physical activity). The category “satisfied with body image” was adopted as reference in the analyses. For multivariate analysis, the variables were included by backward selection process, done according to a certain hierarchy, in blocks. Initially, the variables age, marital status, living conditions, and socioeconomic class were included, and then, BMI, WC, and physical activity. In the final model only those with p≤0.20 remained, and those that presented with a two-tailed value of p<0.05 were considered as having statistical significance. The analyses were carried out with the Stata 11.0 program (Stata Corp., College Station, United States).

## RESULTS

The sociodemographic, economic, anthropometric, physical activity, and body perception characteristics of undergraduate students are presented on table 1. Among the individuals interviewed, 55.7% were male. In both sexes, the highest frequency of undergraduates was within the age range 18-23 years, more than 60.0% resided with their family, most did not live with companions, and belonged to socioeconomic classes A and B.

**Table 1:** Description of the sociodemographic, economic and anthropometric characteristics, physical activity, and body perception of undergraduate students

Variables		Male (n=194)	Female (n=154)
Age, years			
	18-23	89.7 (84.5-93.6)	87.0 (80.6-91.9)
	24-30	10.3 (6.4-15.5)	13.0 (8.1-19.3)
Marital status			
	Lives with partner	40.7 (33.7-48.0)	42.2 (34.3-50.4)
	Does not live with partner	59.3 (52.0-66.2)	57.8 (49.6-65.7)
Living conditions			
	Alone	19.6 (14.2-25.9)	15.6 (10.2-22.3)
	With family	62.9 (55.7-69.7)	66.2 (58.2-73.6)
	In shared housing	17.5 (12.4-23.6)	18.2 (12.4-25.2)
Socioeconomic class			
	A	46.4 (39.2-53.7)	37.7 (30.0-45.8)
	B	39.2 (32.3-46.4)	48.0 (40.0-56.2)
	C, D, and E	14.4 (9.8-20.2)	14.3 (9.2-20.8)
Body mass index			
	Low weight	5.1 (2.5-9.3)	15.6 (10.2-22.3)
	Normal weight	62.4 (55.1-69.2)	69.5 (61.5-76.6)
	Overweight	26.3 (20.2-33.1)	11.7 (7.1-17.8)
	Obesity	6.2 (3.2-10.5)	3.2 (1.1-7.4)
Waist circumference			
	No risk of CVD	91.2 (86.3-94.8)	77.9 (70.5-84.2)
	With risk of CVD	8.8 (5.2-13.6)	22.1 (15.8-29.5)
Physical activity			
	Active	64.4 (57.2-71.1)	59.7 (51.5-67.5)
	Sedentary	35.6 (28.9-42.7)	40.3 (32.4-48.4)
Body perception			
	Satisfied	40.2 (33.2-47.5)	44.8 (36.8-53.0)
	Dissatisfied with excess	32.5 (25.9-39.5)	43.5 (35.5-51.7)
	Dissatisfied with slimness	27.3 (21.2-34.2)	11.7 (7.1-17.8)

Results are expressed as % (95%CI). 95%CI: 95% confidence interval; CVD: cardiovascular disease.

Regarding nutritional status, according to the BMI, 32.5% of men presented with excess weight and 5.1% with low weight. The risk for development of CVD was recorded among 8.8% of them, whereas among women, 14.9% presented with excess weight and 15.6%, with low weight, according to the BMI records. In this group, 22.1% presented with a risk for CVD. Among the population studied, 64.4% of men reported engaging in physical activity, while among women, the frequency of activities was 59.7%. The prevalence of dissatisfaction with body image was 59.8% among men and 55.2% among women.

When comparing the dissatisfaction of body image between men and women, in dissatisfaction due to excess, it was noted that women proved to be more dissatisfied than men − 43.5% and 32.5%, respectively −, while in dissatisfaction due to slimness, men were more dissatisfied (27.3%) than women (11.7%). The difference between men and women was significant (p<0.01).

Tables 2 and 3 display the multinomial logistic regression of dissatisfaction with body image, according to the independent variables for both sexes. For men (Table 2), in the bivariate analysis, age, BMI, and WC were associated with dissatisfaction regarding excess weight. We also noted that the chance of occurrence of dissatisfaction regarding excess was 3.43 times higher among those aged 24-30 years, when compared to those 18-23 years. Men with excess weight presented with a chance of dissatisfaction due to excess weight 8.33 times greater when compared to those who were normal weight. When evaluating the risk for CVD, the chance of occurrence of dissatisfaction due to excess weight was 26.20 times greater among males that presented with the risk when compared with those with no risk for CVD. After the multivariate analysis, BMI and WC remained associated to outcomes. For dissatisfaction due to slimness, no dependent variable was identified by the other variables.

**Table 2:** Multinomial logistic regression of dissatisfaction with body image (reference category: satisfied) and independent variables of male undergraduate students

Variables		Dissatisfaction due to excess weight (n=63)			Dissatisfaction due to slimness (n=53)		
		Prevalence (%)	Bivariate analysis OR (95%CI)	Multivariate analysis OR (95%CI)	Prevalence (%)	Bivariate analysis OR (95%CI)	Multivariate analysis OR (95%CI)
Age, years							
	18-23	29.3	1.00	1.00	28.7	1.00	1.00
	24-30	60.0	3.43 (1.14-10.35)‡	2.64 (0.74-12.26)	15.0	0.87 (0.20-3.83)	0.86 (0.18-4.05)
Marital status							
	Lives with partner	35.4	1.00	1.00	26.6	1.00	1.00
	Does not live with partner	30.4	0.78 (0.39-1.53)	0.76 (0.37-1.54)	27.8	0.95 (0.46-1.94)	0.87 (0.41-1.82)
Living conditions							
	Alone	34.2	1.00	1.00	26.3	1.00	1.00
	With family	30.3	0.88 (0.37-2.09)	0.97 (0.39-2.41)	30.3	1.15 (0.46-2.86)	1.20 (0.47-3.06)
	In shared housing	38.2	1.00 (0.34-2.85)	1.05 (0.34-3.24)	17.6	0.60 (0.17-2.07)	0.57 (0.15-2.04)
Socioeconomic class							
	A	28.9	1.00	1.00	28.9	1.00	1.00
	B	34.2	1.22 (0.59-2.52)	1.15 (0.53-2.51)	25.0	0.89 (0.41-1.91)	0.77 (0.34-1.71)
	C, D, and E	39.3	1.78 (0.64-4.91)	1.27 (0.42-3.84)	28.6	1.29 (0.44-3.80)	1.21 (0.39-3.74)
Body mass index							
	Normal weight	14.9	1.00	1.00	35.5	1.00	1.00
	Low weight	*	†	†	100.0	†	†
	Excess weight	71.4	8.33 (3.89-17.79)‡	5.48 (2.44-12.26)‡	*	†	†
Waist circumference							
	No risk of CVD	26.5	1.00	1.00	29.9	1.00	1.00
	At risk of CVD	94.1	26.20 (3.36-204.07)‡	9.04 (1.09-74.85)‡	*	†	†
Physical activity							
	Active	35.2	1.00	1.00	25.6	1.00	1.00
	Sedentary	27.5	0.72 (0.35-1.48)	1.19 (0.51-2.82)	30.4	1.10 (0.54-2.27)	0.88 (0.40-1.94)

‡ p value <0.05.

* numerical datum equal to zero, not resulting from rounding off;

† null numerical value.

OR: odds ratio; 95%CI: 95% confidence interval; CVD: cardiovascular disease.

**Table 3:** Multinomial logistic regression of dissatisfaction with body image (reference category: satisfied) and independent variables of female undergraduate students

Variables		Dissatisfaction due to excess weight (n=67)			Dissatisfaction due to slimness (n=18)		
		Prevalence (%)	Bivariate analysis OR (95%CI)	Multivariate analysis OR (95%CI)	Prevalence (%)	Bivariate analysis OR (95%CI)	Multivariate analysis OR (95%CI)
Age, years							
	18-23	44.0	1.00	1.00	10.5	1.00	1.00
	24-30	40.0	1.03 (0.36-2.93)	0.89 (0.29-2.71)	20.0	2.17 (0.57-8.26)	1.82 (0.44-7.46)
Marital status							
	Lives with partner	40.0	1.00	1.00	7.7	1.00	1.00
	Does not live with partner	46.1	1.53 (0.77-3.02)	1.38 (0.67-2.85)	16.6	2.52 (0.81-7.85)	2.17 (0.66-7.13)
Living conditions							
	Alone	50.0	1.00	1.00	8.3	1.00	1.00
	With family	36.3	0.60 (0.23-1.54)	0.65 (0.22-1.87)	13.7	1.37 (0.26-7.00)	1.28 (0.19-8.23)
	In shared housing	64.3	1.87 (0.57-6.11)	1.59 (0.45-5.60)	7.1	1.25 (0.14-10.94)	1.39 (0.14-13.85)
Socioeconomic class							
	A	56.9	1.00	1.00	6.9	1.00	1.00
	B	35.1	0.45 (0.21-0.96)‡	0.48 (0.20-1.15)	16.2	1.75 (0.49-6.12)	1.97 (0.48-8.00)
	C, D, and E	36.4	0.42 (0.14-1.21)	0.26 (0.06-1.00)	9.1	0.87 (0.13-5.50)	0.99 (0.14-6.90)
Body mass index							
	Normal weight	42.1	1.00	1.00	7.5	1.00	1.00
	Low weight	4.2	0.09 (0.01-0.73)‡	0.13 (0.01-1.13)	41.7	5.19 (1.71-15.74)‡	4.82 (1.56-14.83)‡
	Excess weight	91.3	12.59 (2.80-56.65)‡	4.15 (0.68-25.36)‡	*	†	†
Waist circumference							
	No risk of CVD	30.0	1.00	1.00	15.0	1.00	1.00
	At risk of CVD	91.2	18.93 (5.41-66.26)‡	9.16 (2.18-38.44)‡	*	†	†
Physical activity							
	Active	45.6	1.00	1.00	11.9	1.00	1.00
	Sedentary	40.3	0.77 (0.38-1.53)	1.13 (0.49-2.59)	11.3	0.82 (0.28-2.38)	0.72 (0.23-2.26)

‡ p value <0.05.

* Numerical datum equal to zero, not resulting from rounding off;

† null numerical value.

OR: odds ratio; 95%CI: 95% confidence interval; CVD: cardiovascular disease.

In the bivariate analysis for women (Table 3), socioeconomic class, BMI, and WC were associated with dissatisfaction due to excess weight. The chance of occurrence of dissatisfaction due to excess was lower (odds ratio, OR=0.45) among women from socioeconomic class B, when compared to those in class A. Among women with excess weight, the chance of occurrence of dissatisfaction due to excess weight was 12.59 times greater when compared to normal-weight women. On the other hand, among those who were underweight, the chance of occurrence of dissatisfaction due to excess weight was lower (OR=0.09) when compared to normal-weight women. According to the WC, women at risk for CVD presented with a chance 18.93 times greater of dissatisfaction due to excess weight relative to those with no risk of CVD. After multivariate analysis, the BMI and WC remained associated to outcomes. For dissatisfaction due to slimness, an association with the BMI was noted, both in bivariate and multivariate analyses. The chance of occurrence of dissatisfaction due to slimness, in the multivariate analysis, was 4.82 times greater among those with low weight when compared to normal-weight women.

## DISCUSSION

This is a young population, at the onset of adult life, in which two-thirds lived with their families, and that generally presented with a good socioeconomic level, similar to that found by other studies investigating body dissatisfaction among undergraduate students.^(^^16^^,^^17^^)^ The nutritional status of the population analyzed was similar to that described by the Research of Family Budgets 2008-2009^(^^18^^)^ for the Brazilian population in this age range, and the frequency of sedentariness is similar to the frequency recorded by the [*Vigilância de Fatores de Risco e Proteção para Doenças Crônicas por Inquérito Telefônico*] (VIGITEL) study [Surveillance of Risk Factors and Protection for Chronic Diseases by Telephone Inquiry].^(^^3^^)^

As to body shapes, historically they have been representations of social status, and over time, these symbols underwent changes. In past times, a high social position was represented by robust and fat bodies, which today is seen as repulsive and a sign of sloppiness, transforming slimness, previously seen as a sign of poverty and disease, into an ideal body to be reached. Thus, all these representations were converted into a reference for feeding behavior. Even though the female body has, comparatively, a greater disposition towards fat and the male body towards muscle, the robust or fat body is not considered attractive. The prominent discourse in the Western culture is “slimness as natural beauty and esthetic ideal.”^(^^4^^)^

This design for a slim and/or muscular body is clearly seen in the results of the present study, since although the smaller part of the men and women evaluated present with a nutritional status outside of standards considered adequate, both by BMI and WC, the majority was dissatisfied with their body image. A comparable standard was also found by Claumann et al.,^(^^16^^)^ among physical education undergraduate students at the *Universidade do Estado de Santa Catarina*, as well as by Rech et al.,^(^^17^^)^ in a study with undergraduate students at the *Universidade Estadual de Ponta Grossa*. The method to evaluate the frequency of body dissatisfaction was the same used in the as the studies mentioned and in the present investigation.

When comparing dissatisfaction with body image between the sexes, it was noted that women are more dissatisfied by excess weight than are men, whereas men are more dissatisfied by slimness when compared to women − albeit, when observing each sex, both have a greater frequency of dissatisfaction due to excess weight, demonstrating the desire to reduce the silhouette. Such results reveal the tendency towards disorders related to the standard of beauty imposed by society, according to which, women are to seek to achieve a standard of beauty strongly associated with slimness, and men, with muscularity.^(^^19^^)^ The search for the standard considered ideal can reflect directly on eating, leading the individuals to seek a negligible consumption of calories or an excess of protein, in order to attain the objective to fitting into the imposed or desired standard.^(^^20^^,^^21^^)^

In addition to changes in eating behavior, body dissatisfaction can lead to the development and maintenance of eating disorders, to low self-esteem, to depression, and to anxiety.^(^^19^^)^ On the other hand, according to Claumann et al.,^(^^16^^)^ and Mintem et al.,^(^^22^^)^ for individuals with excess weight, dissatisfaction with body image can have a positive side, since it is capable of encouraging some individuals to seek a change in appearance, with improved eating and engagement in physical activity. Nonetheless, it is necessary to be attentive to the group of normal-weight people who feel they are above weight, since they could be more inclined to present with a risk behavior.^(^^22^^)^

Just as in the present investigation, other studies with undergraduate students also demonstrated that excess weight is associated with dissatisfaction due to body image.^(^^16^^,^^17^^,^^23^^)^ According to Poltronieri et al.,^(^^24^^)^ results found in a study with women of a municipality in the interior of the state of Rio Grande do Sul, revealed a high prevalence of dissatisfaction with body image (46.0%), regardless of age, besides an association of body dissatisfaction with excess weight, and the presence of symptoms of eating disorders.

Dissatisfaction due to slimness was associated with the nutritional status only among women, and the chance of occurrence of dissatisfaction was about five times greater among those with low weight, according to the BMI, when compared to those who were normal weight. A study on body image in undergraduate students of the State of Sergipe, which also used the scale of nine silhouettes, verified that women were dissatisfied both by being slim and by having excess weight with the same frequency (34.7%), different from this study. The same study indicates that men showed greater dissatisfaction due to slimness (46.2%) than by excess weight (22.7%), while in this study the prevalences of dissatisfaction, both due to excess and due to slimness, were similar among men.^(^^25^^)^

The other variables investigated showed that there was no association with body image after adjusted analysis, suggesting that for the population studied, the frequency of body dissatisfaction does not depend on sociodemographic and economic conditions, or levels of physical activity. The data of the present study are similar to those of other works, which also did not record an association of these variables with the outcome studied.^(^^17^^,^^23^^,^^26^^,^^27^^)^

As a limitation of this study, we should consider that the method utilized to analyze body dissatisfaction in the study does not take into consideration sociocultural aspects, nor does it deepen regarding issues about the desire to lose fat or increase the lean mass, and does not investigate the risk for eating disorders. Nevertheless, it is a low-cost method, with quick application validated in the country; therefore it is valid for investigation of the frequency of body dissatisfaction. Another issue to point out is that many studies that investigate perception of body image are carried out with physical education and nutrition undergraduate students, since this is a public more often subject to receiving information on the body and health. However, in the present study, the sample was composed of undergraduate students from several courses. Therefore, caution is needed when interpreting the data obtained, and the use of other methods together for the evaluation of body image in future studies is recommended, as well as the investigation of dissatisfaction with body image with other variables, such as food consumption, which was not investigated in the present study.

## CONCLUSION

The prevalence of body dissatisfaction was recorded in more than half of the individuals evaluated and was associated to the nutritional status, indicating that the individuals with excess weight and risk of cardiovascular diseases have a greater chance of body dissatisfaction than do those with an adequate nutritional status. However, especially among women, the number of individuals with nutritional status within standards considered normal by the indicators used, but dissatisfied with their body image, stands out.

The findings of the study reinforce the idea that young individuals long for the esthetic ideal of leanness and call attention to the need for public health strategies in order to minimize the pressure exerted by society as to body shapes, and to avoid the possible consequences of body dissatisfaction, such as the inadequate ingestion of nutrients, eating disorders, depression, and anxiety.

## References

[1] 1. Batista Filho M, Rissin A. A transição nutricional no Brasil: tendências regionais e temporais. Cad Saúde Pública. 2003;19(1 suppl 1):181-91. 10.1590/s0102-311x2003000700019 12886448

[2] 2. Popkin BM. Global nutrition dynamics: the world is shifting rapidly toward a diet linked with noncommunicable diseases. Am J Clin Nutr. 2006;84(2):289-98. 10.1093/ajcn/84.1.289 16895874

[3] 3. Brasil. Ministério da Saúde. Secretaria de Vigilância em Saúde.Departamento de Vigilância de Doenças e Agravos não Transmissíveis e Promoção da Saúde. Vigitel Brasil 2016: vigilância de fatores de risco e proteção para doenças crônicas por inquérito telefônico. Brasília (DF): Ministério da Saúde; 2017.

[4] 4. Contreras J, Gracia M. Alimentação, sociedade e cultura. Rio de Janeiro: Editora Fiocruz; 2011. p. 496.

[5] 5. Adami F, Fernandes TC, Frainer DE, Oliveira FR. Aspectos da construção e desenvolvimento da imagem corporal e implicações na Educação Física. Lecturas, Educación Física y Deportes. 2005;83(10).

[6] 6. Pinheiro AP, Giugliani ER. Who are the children with adequate weight who feel fat? J Pediatr (Rio J). 2006;82(3):232-5. 10.2223/JPED.1477 16729150

[7] 7. Graup S, Pereira EF, Lopes AS, Araújo VC, Legnani RF, Borgatto AF. Associação entre a percepção da imagem corporal e indicadores antropométricos de escolares. Rev Bras Educ Fís Esp. 2008;22(2):129-38.

[8] 8. Stunkard AJ, Sørensen T, Schulsinger F. Use of the Danish Adoption Register for the study of obesity and thinness. Res Publ Assoc Res Nerv Ment Dis. 1983;60:115-20. 6823524

[9] 9. Scagliusi FB, Alvarenga M, Polacow VO, Cordás TA, de Oliveira Queiroz GK, Coelho D, et al. Concurrent and discriminant validity of the Stunkard's figure rating scale adapted into Portuguese. Appetite. 2006;47(1):77-82. 10.1016/j.appet.2006.02.010 16750589

[10] 10. Lohman TG, Roche AF, Martorell R. Anthropometric standardization reference manual. Champaign: Human Kinetics; 1988. p.177.

[11] 11. World Health Organization (WHO). Child growth standards. Physical status: the use and the interpretation of an anthropometry: report of a WHO expert committee. Geneva: WHO; 1995. [WHO Tech Rep Series 854]. 8594834

[12] 12. World Health Organization (WHO). Nutrition. Obesity: preventing and managing the global epidemic: report of a WHO Consultation. Geneva: WHO; 2000. p.252. [WHO Techical Report Series 894]. 11234459

[13] 13. Matsudo S, Araújo T, Matsudo V, Andrade D, Andrade E, Oliveira LC, et al. Questionário Internacional de Atividade Física (IPAQ): estudo de validade e reprodutibilidade no Brasil. Ativid Física & Saúde. 2001;6(2):5-18.

[14] 14. World Health Organization (WHO). Global recommendations on physical activity for health. Geneva: WHO; 2010. 26180873

[15] 15. Associação Brasileira de Empresas de Pesquisa (ABEP). Critério de Classificação Econômica Brasil. São Paulo: ABEP; 2014.

[16] 16. Claumann GS, Pereira EF, Inácio S, Santos MC, Martins AC, Pelegrini A. Satisfação com a imagem corporal em acadêmicos ingressantes em cursos de educação física. Rev Educ Fís. 2014;25(4):575-83.

[17] 17. Rech CR, Araújo ED, Vanat JR. Autopercepção da imagem corporal em estudantes do curso de educação física. Rev Bras Educ Fís Esp. 2010; 24(2):285-92.

[18] 18. Instituto Brasileiro de Geografia e Estatística (IBGE). Pesquisa de Orçamentos Familiares 2008-2009: Antropometria e Estado Nutricional de Crianças, Adolescentes e Adultos no Brasil. Rio de Janeiro: IBGE; 2010.

[19] 19. Souza AC, Alvarenga MS. Insatisfação com a imagem corporal em estudantes universitários: uma revisão integrativa. J Bras Psiquiatr. 2016;65(3):286-99.

[20] 20. Souto S, Ferro-Bucher JS. Práticas indiscriminadas de dietas de emagrecimento e o desenvolvimento de transtornos alimentares. Rev Nutri. 2006;19(6): 693-704.

[21] 21. Camargo TP, Costa SP, Uzunian LG, Viebig RF. Vigorexia: revisão dos aspectos atuais deste distúrbio de imagem corporal. Rev Bras Psicol Esporte. 2008;2(1):1-14.

[22] 22. Mintem GC, Gigante DP, Horta BL. Change in body weight and body image in young adults: a longitudinal study. BMC Public Health. 2015;15:222. 10.1186/s12889-015-1579-7 PMC4355136 25879685

[23] 23. Ferrari EP, Petroski EL, Silva DA. Prevalence of body image dissatisfaction and associated factors among physical education students. Trends Psychiatry Psychother. 2013;35(2):119-27. 10.1590/s2237-60892013000200005 25923302

[24] 24. Poltronieri TS, Tusset C, Gregoletto ML, Cremonese C. Insatisfação com a imagem corporal e fatores associados em mulheres do sul do Brasil. Ciên & Saúde. 2016;9(3):128-34.

[25] 25. Silva DA, Nunes HE. Imagem corporal e estágios de mudança de comportamento para atividade física em universitários. Rev Bras Ativ Fís e Saúde. 2014;19(5):597-8.

[26] 26. Silva TR, Saenger G, Pereira EF. Fatores associados à imagem corporal em estudantes de Educação Física. Motriz Rev Educ Fís. 2011;17(4):630-9.

[27] 27. Fortes LS, Miranda VP, Carvalho PH, Ferreira ME. Influências do nível de atividade física e do estado nutricional na insatisfação corporal de universitários de Educação Física. HU Revista. 2011;37(2):175-80.

