# Supplementary material for: Dissatisfaction about body image and associated factors: a study of young undergraduate students
Source: Einstein (Sao Paulo). 2019 Aug 14;17(4):eAO4642. doi: 10.31744/einstein_journal/2019AO4642 (PMC6706222; doi:10.31744/einstein_journal/2019AO4642)
Supplement: Translation

# Translation: Insatisfação da imagem corporal e fatores associados: um estudo em jovens estudantes universitários

**Authors:** Laleska Pâmela Rodrigues da Silva, Ana Rita de Oliveira Tucan, Elisana Lima Rodrigues, Patrícia Vieira Del Ré, Priscila Milene Angelo Sanches, Deise Bresan

**Affiliations:** 1 Faculdade de Ciências Farmacêuticas, Alimentos e Nutrição, Universidade Federal de Mato Grosso do Sul, Campo Grande, MS, Brasil

**Keywords:** Autoimagem, Estado nutricional, Imagem corporal, Peso corporal, Estudantes

## Abstract

**Objetivo:**

Verificar a prevalência de insatisfação com a imagem corporal e sua associação com variáveis sociodemográficas, econômicas, antropométricas e atividade física de universitários.

**Métodos:**

Estudo transversal, realizado com 348 universitários que frequentavam o restaurante universitário de uma universidade pública no Estado do Mato Grosso do Sul. A percepção da imagem corporal foi avaliada pelo *Silhouette Matching Task*. As demais variáveis avaliadas foram sexo, idade, condição marital, condição de moradia, classe econômica, peso, estatura, circunferência da cintura e nível de atividade física. Para verificar a associação entre as variáveis dependente e independente, foi realizada regressão logística multinomial.

**Resultados:**

Dentre os entrevistados, 55,7% eram homens. A prevalência de insatisfação com a imagem corporal foi de 59,8% entre os homens e de 55,2% entre as mulheres. A insatisfação pelo excesso de peso, entre homens e mulheres, foi maior nos indivíduos com excesso de peso, quando comparados aos eutróficos, segundo o índice de massa corporal, e também maior naqueles com risco para doenças cardiovasculares, quando comparados aos que não apresentaram risco. A insatisfação pela magreza foi maior entre as mulheres com baixo peso quando comparadas às eutróficas, segundo o índice de massa corporal. Entre os homens, não houve associação da insatisfação com a magreza e as variáveis estudadas.

**Conclusão:**

A prevalência da insatisfação corporal foi registrada em mais da metade dos indivíduos avaliados e esteve associada ao estado nutricional. Sabendo das consequências da insatisfação corporal, vale destacar a necessidade de estratégias de intervenção, no sentido de evitar a prática de condutas comportamentais não saudáveis.

## INTRODUÇÃO

A partir da metade do século 20, no Brasil, começaram a ser observadas mudanças no perfil de morbimortalidade da população. Transformações nos padrões alimentares e de atividade física têm sido, em partes, responsáveis pelo processo de transição nutricional na população brasileira, com a diminuição da desnutrição e o aumento da prevalência de sobrepeso e obesidade entre os adultos.^(^^1^^,^^2^^)^ Atualmente, metade dos adultos no país apresenta excesso de peso, e 18,9% são obesos.^(^^3^^)^

Ao mesmo tempo em que se experimenta uma sociedade dos excessos, na qual muitos produtos alimentícios estão disponíveis para compra, com grande disponibilidade de alimentos processados e ultraprocessados, paradoxalmente se convive com milhares de mensagens para evitá-los, em uma sociedade que se promovem a satisfação perpétua e a magreza mais rigorosa.^(^^4^^)^ A pressão exercida pelos discursos dietéticos e pelo *marketing* do corpo na construção da imagem corporal é desmedida, e, nas últimas décadas, um número cada vez maior de pessoas vem manifestando insatisfação com suas formas corporais, abstendo-se de determinados alimentos e utilizando mecanismos muitas vezes inapropriados para perda de peso.^(^^4^^)^

A percepção da imagem corporal é um complexo fenômeno que envolve aspectos cognitivos, afetivos, sociais e culturais. É influenciada pelas dinâmicas interações entre o ser e o meio em que ele vive, sendo baseada nos sentimentos que uma pessoa possui em relação ao tamanho e às formas corporais.^(^^5^^)^ Os padrões de beleza, fortemente influenciados pela mídia e fortalecidos pela sociedade, têm exigido perfis antropométricos cada vez mais magros, levando, muitas vezes, à preocupação com a imagem corporal, mesmo com estado nutricional dentro dos parâmetros considerados adequados para faixa etária e sexo.^(^^6^^,^^7^^)^

Dessa maneira, é fundamental conhecer a frequência da insatisfação corporal na população brasileira e quais os possíveis fatores associados, na medida em que os dados produzidos podem servir de base para futuras políticas e programas na área da saúde, afim de incentivar hábitos alimentares saudáveis e minimizar a pressão exercida pela sociedade relacionada ao corpo, visando prevenir as consequências desencadeadas pela insatisfação.

## OBJETIVO

Verificar a prevalência de insatisfação com a imagem corporal e sua associação com variáveis sociodemográficas, econômicas, antropométricas e atividade física de universitários de ambos os sexos.

## MÉTODOS

Trata-se de estudo transversal, realizado com 348 acadêmicos que frequentavam o restaurante universitário de uma universidade pública do Estado do Mato Grosso do Sul. Para o cálculo da amostra, foi utilizado o *software* OpenEpi (versão 3.0.1), com população-alvo de 1.800 indivíduos (média diária de comensais), prevalência esperada de 50,0%, margem de erro de 5 pontos percentuais e erro alfa de 5,0%. Os acadêmicos foram selecionados na fila do restaurante por amostragem sistemática (a cada 15 indivíduos, selecionou-se o décimo quinto). Os critérios de inclusão foram: frequentar o restaurante pelo menos três vezes na semana, ter idade igual ou superior a 18 anos e inferior a 30 anos, e não estar gestante. A pesquisa foi aprovada pelo Comitê de Ética em Pesquisa em Seres Humanos (parecer 867. 367, CAAE: 37575114.1.0000.0021), e todos os participantes assinaram o Termo de Consentimento Livre e Esclarecido (TCLE). A coleta de dados foi realizada durante 5 dias consecutivos no mês de novembro de 2015, por uma equipe previamente treinada, e após a execução de um projeto piloto. Foram coletados dados sobre percepção da imagem corporal, estado nutricional, atividade física, classe socioeconômica e variáveis sociodemográficas (sexo, idade, condição de moradia e condição marital).

Para avaliação da percepção da imagem corporal, foi utilizada a *Silhouette Matching Task* (SMT) ou Teste de Avaliação da Imagem Corporal,^(^^8^^)^ adaptada e validada para adultos brasileiros,^(^^9^^)^ que consiste em uma escala com nove figuras de silhuetas visuais. O indivíduo foi orientado a escolher uma das figuras que correspondesse à sua silhueta corporal autoavaliada e outra à silhueta corporal desejada. Quando houve diferença entre as silhuetas escolhidas, o indivíduo foi considerado insatisfeito com a imagem corporal. A insatisfação pelo excesso ocorreu quando a silhueta desejada foi menor que a autoavaliada e a insatisfação pela magreza quando a silhueta desejada foi maior que a autoavaliada.

Para determinação do estado nutricional, foram coletadas as seguintes variáveis: peso corporal, estatura e circunferência da cintura (CC). O peso corporal, a estatura e a CC foram aferidos segundo o protocolo de Lohman et al.,^(^^10^^)^ Para a medida de peso, foi utilizada balança digital portátil, do tipo plataforma, com capacidade para 150kg e precisão de 100g. A estatura foi aferida com fita métrica flexível e inextensível, com precisão de 1mm, esta foi fixada na parede com altura a partir de 50cm. A CC foi aferida com trena metálica flexível e inextensível, com precisão de 1mm. Após a obtenção dos dados de peso corporal e estatura, foi calculado o índice de massa corporal (IMC) e classificado segundo os pontos de corte proposto pela Organização Mundial da Saúde (OMS).^(^^11^^)^ A CC foi classificada segundo critérios propostos pela OMS.^(^^12^^)^

O nível de atividade física foi avaliado pelo Questionário Internacional de Atividade Física (IPAQ), versão curta.^(^^13^^)^ A classificação seguiu os critérios da OMS, que considera como praticante de atividade física aquele indivíduo que realiza pelo menos 150 minutos por semana de atividade física moderada ou 75 minutos de atividade física vigorosa, em sessões de pelo menos 10 minutos de duração, sem determinação de frequência semanal.^(^^14^^)^

Para determinação da classe socioeconômica, foi aplicado o Critério de Classificação Econômica Brasil, da Associação Brasileira de Empresas de Pesquisa (ABEP).^(^^15^^)^

Os dados foram tabulados no EpiData 3.1 (EpiData Assoc., Odense, Dinamarca). A estatística descritiva foi realizada por frequência relativa e intervalo de confiança de 95% (IC95%). Para verificar a associação entre a variável dependente (percepção da imagem corporal categorizada em satisfeitos com a imagem corporal, insatisfeitos pelo excesso de peso e insatisfeitos pela magreza) e as variáveis independentes (idade, condição marital, condição de moradia, classe socioeconômica, IMC, CC e atividade física), foi realizada regressão logística multinomial. A categoria “satisfeitos com a imagem corporal” foi adotada como referência nas análises. Para análise multivariada, as variáveis foram incluídas por processo de seleção para trás (*backward*), realizado segundo determinada hierarquia, em blocos. Inicialmente, foram incluídas as variáveis idade, condição marital, condição de moradia e classe socioeconômica e, na sequência, IMC, CC e atividade física. Permaneceram no modelo final apenas aquelas com p≤0,20, e foram consideradas com significância estatística aquelas que apresentaram valor de p<0,05 bicaudais. As análises foram realizadas no programa Stata 11.0 (Stata Corp., College Station, Estados Unidos).

## RESULTADOS

As características sociodemográficas, econômicas, antropométricas, atividade física e percepção corporal de universitários estão apresentadas na tabela 1. Entre os indivíduos entrevistados, 55,7% eram do sexo masculino. Em ambos os sexos, a maior frequência de universitários esteve na faixa etária de 18 a 23 anos, mais de 60,0% residiam com a família, e a maior parte não residia com companheiros e pertencia às classes socioeconômicas A e B.

**Tabela 1:** Descrição das características sociodemográficas, econômicas, antropométricas, atividade física e percepção corporal de universitários

Variáveis		Masculino (n=194)	Feminino (n=154)
Idade, anos			
	18-23	89,7 (84,5-93,6)	87,0 (80,6-91,9)
	24-30	10,3 (6,4-15,5)	13,0 (8,1-19,3)
Condição marital			
	Reside com companheiro	40,7 (33,7-48,0)	42,2 (34,3-50,4)
	Não reside com companheiro	59,3 (52,0-66,2)	57,8 (49,6-65,7)
Condição de moradia			
	Sozinho	19,6 (14,2-25,9)	15,6 (10,2-22,3)
	Com família	62,9 (55,7-69,7)	66,2 (58,2-73,6)
	Em república	17,5 (12,4-23,6)	18,2 (12,4-25,2)
Classe socioeconômica			
	A	46,4 (39,2-53,7)	37,7 (30,0-45,8)
	B	39,2 (32,3-46,4)	48,0 (40,0-56,2)
	C, D e E	14,4 (9,8-20,2)	14,3 (9,2-20,8)
Índice de massa corporal			
	Baixo peso	5,1 (2,5-9,3)	15,6 (10,2-22,3)
	Eutrofia	62,4 (55,1-69,2)	69,5 (61,5-76,6)
	Sobrepeso	26,3 (20,2-33,1)	11,7 (7,1-17,8)
	Obesidade	6,2 (3,2-10,5)	3,2 (1,1-7,4)
Circunferência da cintura			
	Sem risco de DCV	91,2 (86,3-94,8)	77,9 (70,5-84,2)
	Com risco de DCV	8,8 (5,2-13,6)	22,1 (15,8-29,5)
Atividade física			
	Praticante	64,4 (57,2-71,1)	59,7 (51,5-67,5)
	Sedentário	35,6 (28,9-42,7)	40,3 (32,4-48,4)
Percepção corporal			
	Satisfeito	40,2 (33,2-47,5)	44,8 (36,8-53,0)
	Insatisfeito excesso	32,5 (25,9-39,5)	43,5 (35,5-51,7)
	Insatisfeito magreza	27,3 (21,2-34,2)	11,7 (7,1-17,8)

Resultados expressos como % (IC95%). IC95%: intervalo de confiança de 95%; DCV: doença cardiovascular.

No que se refere ao estado nutricional, segundo o IMC, 32,5% dos homens apresentaram excesso de peso e 5,1% estavam baixo peso. O risco para desenvolvimento de DCV foi registrado entre 8,8% deles. Já entre as mulheres, 14,9% apresentaram excesso de peso e 15,6%, baixo peso, de acordo como o registro do IMC. Nesse grupo, 22,1% apresentaram risco para DCV. Entre a população estudada, 64,4% dos homens referiram praticar atividade física, enquanto que, entre as mulheres, a frequência de praticantes foi 59,7%. A prevalência de insatisfação com a imagem corporal foi de 59,8% entre os homens e 55,2% entre as mulheres.

Ao comparar a insatisfação da imagem corporal entre homens e mulheres, notou-se que, na insatisfação pelo excesso, as mulheres se mostraram mais insatisfeitas que os homens − 43,5% e 32,5%, respectivamente −, enquanto na insatisfação pela magreza, os homens estavam mais insatisfeitos (27,3%) do que as mulheres (11,7%). A diferença entre homens e mulheres foi significativa (p<0,01).

As tabelas 2 e 3 apresentam a regressão logística multinomial da insatisfação com a imagem corporal, segundo as variáveis independentes para ambos os sexos. Para os homens (Tabela 2), na análise bivariada, idade, IMC e CC estiveram associados com a insatisfação pelo excesso de peso. Observou-se que a chance de ocorrência de insatisfação pelo excesso foi 3,43 vezes maior entre aqueles que possuíam 24 a 30 anos de idade quando comparados aos que possuíam de 18 a 23 anos. Os homens com excesso de peso apresentaram chance de insatisfação pelo excesso de peso 8,33 vezes maior quando comparados aos eutróficos. Ao avaliar o risco para DCV, a chance de ocorrência de insatisfação pelo excesso de peso foi 26,20 vezes maior entre os homens que apresentam o risco quando comparados aqueles sem risco para DCV. Após a análise multivariada, permaneceram associados ao desfecho o IMC e a CC. Para a insatisfação pela magreza, não foi identificada associação da variável dependente com as demais variáveis.

**Tabela 2:** Regressão logística multinomial da insatisfação com a imagem corporal (categoria de referência: satisfeitos) e variáveis independentes de universitários do sexo masculino

Variáveis		Insatisfação pelo excesso de peso (n=63)			Insatisfação pela magreza (n=53)		
		Prevalência (%)	Análise bivariada OR (IC95%)	Análise multivariada OR (IC95%)	Prevalência (%)	Análise bivariada OR (IC95%)	Análise multivariada OR (IC95%)
Idade, anos							
	18-23	29,3	1,00	1,00	28,7	1,00	1,00
	24-30	60,0	3,43 (1,14-10,35)‡	2,64 (0,74-12,26)	15,0	0,87 (0,20-3,83)	0,86 (0,18-4,05)
Condição marital							
	Reside com companheiro	35,4	1,00	1,00	26,6	1,00	1,00
	Não reside com companheiro	30,4	0,78 (0,39-1,53)	0,76 (0,37-1,54)	27,8	0,95 (0,46-1,94)	0,87 (0,41-1,82)
Condição de moradia							
	Sozinho	34,2	1,00	1,00	26,3	1,00	1,00
	Com família	30,3	0,88 (0,37-2,09)	0,97 (0,39-2,41)	30,3	1,15 (0,46-2,86)	1,20 (0,47-3,06)
	Em república	38,2	1,00 (0,34-2,85)	1,05 (0,34-3,24)	17,6	0,60 (0,17-2,07)	0,57 (0,15-2,04)
Classe socioeconômica							
	A	28,9	1,00	1,00	28,9	1,00	1,00
	B	34,2	1,22 (0,59-2,52)	1,15 (0,53-2,51)	25,0	0,89 (0,41-1,91)	0,77 (0,34-1,71)
	C, D e E	39,3	1,78 (0,64-4,91)	1,27 (0,42-3,84)	28,6	1,29 (0,44-3,80)	1,21 (0,39-3,74)
Índice de massa corporal							
	Eutrofia	14,9	1,00	1,00	35,5	1,00	1,00
	Baixo peso	*	†	†	100,0	†	†
	Excesso de peso	71,4	8,33 (3,89-17,79)‡	5,48 (2,44-12,26)‡	*	†	†
Circunferência da cintura							
	Sem risco de DCV	26,5	1,00	1,00	29,9	1,00	1,00
	Com risco de DCV	94,1	26,20 (3,36-204,07)‡	9,04 (1,09-74,85)‡	*	†	†
Atividade física							
	Praticante	35,2	1,00	1,00	25,6	1,00	1,00
	Sedentário	27,5	0,72 (0,35-1,48)	1,19 (0,51-2,82)	30,4	1,10 (0,54-2,27)	0,88 (0,40-1,94)

‡ valor de p<0,05.

* Dado numérico igual a zero, não resultante de arredondamento;

† valor numérico nulo.

OR: *odds ratio*; IC95%: intervalo de confiança de 95%; DCV: doença cardiovascular.

**Tabela 3:** Regressão logística multinomial da insatisfação com a imagem corporal (categoria de referência: satisfeitas) e variáveis independentes de universitários do sexo feminino

Variáveis		Insatisfação pelo excesso de peso (n=67)			Insatisfação pela magreza (n=18)		
		Prevalência (%)	Análise bivariada OR (IC95%)	Análise multivariada OR (IC95%)	Prevalência (%)	Análise bivariada OR (IC95%)	Análise multivariada OR (IC95%)
Idade, anos							
	18-23	44,0	1,00	1,00	10,5	1,00	1,00
	24-30	40,0	1,03 (0,36-2,93)	0,89 (0,29-2,71)	20,0	2,17 (0,57-8,26)	1,82 (0,44-7,46)
Condição marital							
	Reside com companheiro	40,0	1,00	1,00	7,7	1,00	1,00
	Não reside com companheiro	46,1	1,53 (0,77-3,02)	1,38 (0,67-2,85)	16,6	2,52 (0,81-7,85)	2,17 (0,66-7,13)
Condição de moradia							
	Sozinho	50,0	1,00	1,00	8,3	1,00	1,00
	Com família	36,3	0,60 (0,23-1,54)	0,65 (0,22-1,87)	13,7	1,37 (0,26-7,00)	1,28 (0,19-8,23)
Em república		64,3	1,87 (0,57-6,11)	1,59 (0,45-5,60)	7,1	1,25 (0,14-10,94)	1,39 (0,14-13,85)
	Classe socioeconômica						
	A	56,9	1,00	1,00	6,9	1,00	1,00
	B	35,1	0,45 (0,21-0,96)‡	0,48 (0,20-1,15)	16,2	1,75 (0,49-6,12)	1,97 (0,48-8,00)
	C, D e E	36,4	0,42 (0,14-1,21)	0,26 (0,06-1,00)	9,1	0,87 (0,13-5,50)	0,99 (0,14-6,90)
Índice de massa corporal							
	Eutrofia	42,1	1,00	1,00	7,5	1,00	1,00
	Baixo peso	4,2	0,09 (0,01-0,73)‡	0,13 (0,01-1,13)	41,7	5,19 (1,71-15,74)‡	4,82 (1,56-14,83)‡
	Excesso de peso	91,3	12,59 (2,80-56,65)‡	4,15 (0,68-25,36)‡	*	†	†
Circunferência da cintura							
	Sem risco de DCV	30,0	1,00	1,00	15,0	1,00	1,00
	Com risco de DCV	91,2	18,93 (5,41-66,26)‡	9,16 (2,18-38,44)‡	*	†	†
Atividade física							
	Praticante	45,6	1,00	1,00	11,9	1,00	1,00
	Sedentário	40,3	0,77 (0,38-1,53)	1,13 (0,49-2,59)	11,3	0,82 (0,28-2,38)	0,72 (0,23-2,26)

‡ valor de p<0,05.

* Dado numérico igual a zero, não resultante de arredondamento;

† valor numérico nulo.

OR: *odds ratio*; IC95%: intervalo de confiança de 95%; DCV: doença cardiovascular.

Para as mulheres (Tabela 3), na análise bivariada, classe socioeconômica, IMC e CC se associaram com a insatisfação peso excesso de peso. A chance de ocorrência de insatisfação pelo excesso foi menor (*odds ratio*, OR=0,45) entre as mulheres pertencentes a classe socieoconômica B quando comparadas àquelas pertencentes à classe A. Entre as mulheres com excesso de peso, a chance de ocorrência de insatisfação pelo excesso de peso foi 12,59 vezes maior quando comparadas às mulheres em estado de eutrofia. Já entre aquelas com baixo peso, a chance de ocorrência de insatisfação pelo excesso foi menor (OR=0,09) quando comparadas às eutróficas. Segundo a CC, as mulheres com risco para DCV apresentaram chance 18,93 vezes maior de insatisfação pelo excesso de peso com relação àquelas sem risco para DCV. Após análise multivariada permaneceram associados ao desfecho o IMC e a CC. Para insatisfação pela magreza, foi observada associação com o IMC, tanto na análise bivariada como na multivariada. A chance de ocorrência de insatisfação pela magreza, na análise multivariada, foi 4,82 vezes maior entre aquelas com baixo peso quando comparadas às eutróficas.

## DISCUSSÃO

Trata-se de população jovem, no início da fase adulta, da qual dois terços residiam com a família, e que apresentava, em geral, bom nível socioeconômico, semelhante ao encontrado por outros estudos que investigaram a insatisfação corporal entre universitários.^(^^16^^,^^17^^)^ O estado nutricional da população avaliada foi semelhante àquele descrito pela Pesquisa de Orçamentos Familiares 2008-2009^(^^18^^)^ para a população brasileira nessa faixa etária, assim como a frequência de indivíduos sedentários se assemelha à frequência registrada pelo estudo de Vigilância de Fatores de Risco e Proteção para Doenças Crônicas por Inquérito Telefônico (VIGITEL).^(^^3^^)^

No que se refere às formas corporais, historicamente elas foram representações de *status* e, com o passar dos séculos, esses símbolos sofreram mudanças. Em épocas passadas, a alta posição social era representada por corpos robustos e gordos, o que hoje é visto com repulsa e sinal de desleixo, transformando a magreza, anteriormente vista como sinal de pobreza e doença, em um ideal de corpo a ser alcançado e convertendo, assim, todas essas representações em um norte para o comportamento alimentar. Ainda que o corpo feminino tenha, comparativamente, maior predisposição à gordura e o masculino ao músculo, o corpo robusto ou gordo não é considerado belo. O discurso predominante na cultura ocidental é a “magreza como beleza natural e ideal estético”.^(^^4^^)^

Esse desejo por um corpo magro e/ou musculoso é claramente visto nos resultados do presente estudo, pois, embora a menor parte dos homens e mulheres avaliados apresente estado nutricional fora dos padrões considerados adequados, tanto pelo IMC como pela CC, a maior parte estava insatisfeita com sua imagem corporal. Padrão parecido foi também encontrado por Claumann et al.,^(^^16^^)^ entre estudantes de educação física da Universidade do Estado de Santa Catarina, assim como por Rech et al.,^(^^17^^)^ em estudo com universitários da Universidade Estadual de Ponta Grossa. O método para avaliar a frequência de insatisfação corporal foi o mesmo nos estudos citados e na presente pesquisa.

Ao comparar a insatisfação com a imagem corporal entre os sexos, observou-se que as mulheres são mais insatisfeitas pelo excesso de peso do que os homens, enquanto que os homens são mais insatisfeitos pela magreza quando comparados às mulheres − embora, ao observar cada sexo, ambos tenham frequência maior de insatisfação pelo excesso de peso, demonstrando o desejo de reduzir a silhueta. Tais resultados revelam tendência a transtornos relacionados com o padrão de beleza imposto pela sociedade, segundo o qual é colocado às mulheres um ideal de beleza fortemente associado à magreza e, aos homens, à muscularidade.^(^^19^^)^ A busca pelo padrão considerado ideal pode refletir diretamente na alimentação, fazendo com que os indivíduos busquem um consumo ínfimo de calorias ou excesso de proteína, para atingir o objetivo de se enquadrar/adequar ao padrão imposto e desejado.^(^^20^^,^^21^^)^

Além de mudanças no comportamento alimentar, a insatisfação corporal pode levar ao desenvolvimento e manutenção de transtornos alimentares, à baixa autoestima, à depressão e à ansiedade.^(^^19^^)^ Por outro lado, segundo Claumann et al.,^(^^16^^)^ e Mintem et al.,^(^^22^^)^ para indivíduos com excesso de peso, a insatisfação com a imagem corporal pode ter um lado positivo, na medida em que é capaz de estimular alguns indivíduos a buscarem a mudança na aparência, com melhora alimentar e prática de atividade física. Porém, é preciso estar atento ao grupo de eutróficos que se sentem acima do peso, uma vez que ele pode estar mais propenso a apresentar comportamento de risco.^(^^22^^)^

Assim como na presente pesquisa, outros estudos com universitários também demonstraram que o excesso de peso está associado à insatisfação com a imagem corporal.^(^^16^^,^^17^^,^^23^^)^ De acordo com Poltronieri et al.,^(^^24^^)^ resultados encontrados em estudo com mulheres de um município do interior do Rio Grande do Sul, revelaram alta prevalência de insatisfação com a imagem corporal (46,0%), independente da idade, além da associação da insatisfação corporal com o excesso de peso e a presença de sintomas de transtornos alimentares.

A insatisfação pela magreza esteve associada ao estado nutricional apenas entre as mulheres, e a chance de ocorrência de insatisfação foi cerca de cinco vezes maior entre aquelas com baixo peso, segundo o IMC, quando comparadas àquelas com eutrofia. Estudo sobre a imagem corporal em universitários do Estado de Sergipe, que também utilizou a escala de nove silhuetas, verificou que as mulheres se encontravam insatisfeitas tanto pela magreza como pelo excesso, com a mesma frequência (34,7%), diferente do presente estudo. O mesmo estudo indica que os homens apresentam maior insatisfação pela magreza (46,2%) do que pelo excesso (22,7%), enquanto neste estudo as prevalências de insatisfação, tanto pelo excesso como pela magreza, foram semelhantes entre os homens.^(^^25^^)^

As demais variáveis investigadas não apresentaram associação com a imagem corporal após a análise ajustada, sugerindo que, para a população estudada, a frequência de insatisfação corporal independe das condições sociodemográficas, econômicas e níveis de atividade física. Os dados do presente estudo vão ao encontro de outros trabalhos, que também não registraram associação dessas variáveis com o desfecho estudado.^(^^17^^,^^23^^,^^26^^,^^27^^)^

Como limitação do presente estudo, devemos considerar que o método utilizado para avaliar a insatisfação corporal no presente estudo não leva em conta aspectos socioculturais, nem mesmo se aprofunda em questões sobre o desejo de perder gordura ou aumentar massa magra, e também não investiga os riscos para transtornos alimentares. No entanto, trata-se de um método de rápida aplicação, validado no país, com baixo custo e que, assim, é válido para investigação da frequência da insatisfação corporal. Outro ponto a ser destacado é que grande parte dos estudos que investigam a percepção da imagem corporal são realizados com acadêmicos de educação física e nutrição, tratando-se de um público que está mais sujeito a receber informações sobre corpo e saúde, enquanto que, no atual estudo, a amostra foi composta por universitários de diversos cursos. Portanto, é necessária cautela ao se interpretarem os dados obtidos, recomendando-se a utilização de outros métodos em conjunto para a avaliação da imagem corporal em futuros estudos, assim como a investigação da associação da insatisfação com a imagem corporal com outras variáveis, como o consumo alimentar, não investigado no presente estudo.

## CONCLUSÃO

A prevalência da insatisfação corporal foi registrada em mais da metade dos indivíduos avaliados e esteve associada ao estado nutricional, indicando que os indivíduos com excesso de peso e risco para doenças cardiovasculares têm chance de ocorrência de insatisfação corporal maior do que aqueles com estado nutricional adequado. Contudo, principalmente entre as mulheres, chama a atenção o número de indivíduos com estado nutricional dentro dos padrões considerados normais, pelos indicadores utilizados, e insatisfeitos com a imagem corporal.

Os achados do estudo reforçam a ideia de que indivíduos jovens anseiam o ideal estético da magreza e alertam para necessidade de estratégias em saúde pública, a fim de minimizar a pressão exercida pela sociedade com relação às formas corporais, e evitar as possíveis consequências da insatisfação corporal, como a ingestão inadequada de nutrientes, transtornos alimentares, depressão e ansiedade.
